# Assessment of the Cytotoxicity, Mutagenicity, and Genotoxicity of Two Traditional Chinese Herbs: *Aristolochia baetica* and *Magnolia officinalis*

**DOI:** 10.3390/toxins15010052

**Published:** 2023-01-06

**Authors:** Mélanie Poivre, Marie-Hélène Antoine, Kirill Kryshen, Anastasia Atsapkina, Alexander N. Shikov, Laure Twyffels, Amandine Nachtergael, Pierre Duez, Joëlle Nortier

**Affiliations:** 1 Laboratory of Experimental Nephrology, Faculty of Medecine, Université Libre de Bruxelles, 1000 Bruxelles, Belgium; 2 Saint-Petersburg Institute of Pharmacy, 197376 Saint Petersburg, Russia; 3 Laboratory of Therapeutic Chemistry and Pharmacognosy, Faculty of Medicine and Pharmacy, Research Institute for Health Sciences and Technology, University of Mons—UMONS, 7000 Mons, Belgium; 4 Department of Technology of Pharmaceutical Formulations, St. Petersburg State Chemical Pharmaceutical University, Prof. Popov 14a, 197376 Saint Petersburg, Russia; 5 CMMI Center for Microscopy and Molecular Imaging, 6041 Charleroi, Belgium

**Keywords:** *Aristolochia*, *Magnolia*, mutagenicity, genotoxicity, cytotoxicity, Ames test, γH2AX

## Abstract

Herbal remedies used in traditional medicine often contain several compounds combined in order to potentiate their own intrinsic properties. However, herbs can sometimes cause serious health troubles. In Belgium, patients who developed severe aristolochic acid nephropathy ingested slimming pills containing root extracts of an *Aristolochia* species, as well as the bark of *Magnolia officinalis*. The goal of the study was to evaluate, on a human renal cell line, *Aristolochia* and *Magnolia* extracts for their cytotoxicity by a resazurin cell viability assay, and their genotoxicity by immunodetection and quantification of the phosphorylated histone γ-H2AX. The present study also sought to assess the mutagenicity of these extracts, employing an OECD recognized test, the Ames test, using four *Salmonella typhimurium* strains with and without a microsomial fraction. Based on our results, it has been demonstrated that the *Aristolochia–Magnolia* combination (aqueous extracts) was more genotoxic to human kidney cells, and that this combination (aqueous and methanolic extracts) was more cytotoxic to human kidney cells after 24 and 48 h. Interestingly, it has also been shown that the *Aristolochia–Magnolia* combination (aqueous extracts) was mutagenic with a TA98 *Salmonella typhimurium* strain in the presence of a microsomial liver S9 fraction. This mutagenic effect appears to be dose-dependent.

## 1. Introduction

Following disappointment with allopathic treatments, patients may turn to other therapies such as phytotherapy. Phytotherapy is increasingly considered by the general public as a safe and reliable therapy. Moreover, plant derivatives (such as *Magnolia officinalis* or herbal medicinal products that contain aristolochic acids) are often readily available either in supermarkets or on the internet, and sometimes the products’ degree of purity is not suitable for pharmaceutical use [1]. For example, mycotoxins, mainly *Fusarium* toxins, are naturally present in several plants that are widely used as feedstuffs worldwide [2,3]. Although often perceived as innocuous by the general public, many herbs harbor phytochemicals that are either directly reactive towards DNA, or likely to disturb cellular homoeostasis, cell cycle, and/or genome maintenance mechanisms; this may translate into genotoxicity, carcinogenicity, or co-carcinogenicity [4]. When taken orally (in the form of pills, tea, syrup, etc.), plants are excreted by the kidneys. Indeed, the excretory organs, such as the kidney and bladder, are often the sites of toxicity. Moreover, kidney diseases often lead to an increased risk of morbidity from cardiovascular diseases [5,6].

Genotoxicity refers to the deleterious effect of a chemical compound or a physical event on the genetic material; such genotoxic events are considered hallmarks of cancer risk [4].

In the early 1990s, an epidemic of rapidly progressive tubulointerstitial nephritis was reported in young Belgian female patients who inadvertently took slimming pills containing the bark of *Magnolia officinalis* Rehder & E. H. Wilson, and inadvertently the roots of an *Aristolochia* species [7,8,9].

At that time, the causative nephrotoxic agent was identified as aristolochic acid (AA), and the renal disease following their ingestion is now worldwide recognized as Aristolochic Acid Nephropathy (AAN) [10,11]. Remarkably, despite the worldwide human consumption of AA, the *Materia Medica* and *Pharmacopoeia* before 1995 failed to mention any of the toxic effects of *Aristochia* spp. [12].

AA was classified as a human carcinogen Class I by the World Health Organization International Agency for Research on Cancer in 2002 [13]. The nephrotoxic effect of aristolochic acid is irreversible. AAN and associated upper urinary tract urothelial carcinoma and bladder cancer may become a major public health threat in the next few years [4,14].

However, despite *Aristolochia* species prohibition in many countries, more cases of AA intoxication have been reported all over the world, especially in Asia and in Balkan countries [9,15]. For example, there is accumulating evidence that Balkan endemic nephropathy (BEN) is an environmental disease caused by AAs released from the decomposition of *Aristolochia clematitis* L., an AA-containing weed that grows abundantly in the Balkan Peninsula [16,17].

Nowadays, herbal medicinal products that contain AA continue to be manufactured and marketed worldwide with inadequate regulation, and possible environmental exposure routes receive little attention [1].

In 2015, Nachtergael et al. showed the effect of *M. officinalis* on *Aristolochia* genotoxicity. The high potentiation of AA genotoxicity by *M. officinalis* can be tentatively explained by an increased metabolic activation into aristolactams [18].

As summarized in Table 1, major significant bioactive components isolated from the bark of *M. officinalis* appear to be the polyphenolic neolignans, magnolol, and honokiol [19,20]. Different tertiary and quaternary alkaloids have also been isolated and structurally elucidated [20], including (i) the aporphine alkaloids *N*-methylcoxylonine, (*S*)-magnoflorine, magnofficine, (*R*)-asimilobine, corytubérine, anonaine, and liriodenine and (ii) benzyltetrahydroisoquinoline alkaloids (*R*)-magnocurarine, (*S*)-tembetarine, lotusine, (*R*)-oblongine, and reticuline [21,22]. Magnoflorine and magnocurarine are considered the major and most potent *Magnolia* bark alkaloids [23].

In the present study, *Magnolia* effects in the AAN case were further investigated. Following the nephrotoxicity of AA compounds, studies were performed in HK-2 kidney proximal human tubule cells.

The goal of the present study was to evaluate, on a renal cell line, *Aristolochia* and *Magnolia* aqueous and methanolic extracts, alone and in combination, for their cytotoxicity by a resazurine cell viability assay, and their genotoxicity by immunodetection and quantification of the phosphorylated histone γ-H2AX, a genotoxicity biomarker [24,25]. The present study also sought to assess the mutagenicity of these compounds, using an OECD recognized test, the Ames test, using four *Salmonella typhimurium* strains—TA98, TA100, TA1535, and TA1537—with and without the S9 microsomal fraction, often used to mimic mammal metabolism and to estimate the need for bioactivation [26].

## 2. Results

### 2.1. Plant Extract Characterization

#### 2.1.1. *Aristolochia baetica* L.

The thin-layer chromatography of raw plant material and the aqueous extract of *A. baetica* was used according to the European Pharmacopoeia 8.4 monograph “Test for aristolochic acids in herbal drugs,” previously detailed by Nachtergael et al. In brief, the following was used: an HPTLC Silica gel 60 plate F254, a mobile phase of anhydrous formic acid, water, ethyl acetate, and toluene (3:3:30:60, *v*/*v*/*v*/*v*), an upper layer detection method using 100 g/L Tin(II) chloride in diluted hydrochloric acid at 100 °C for 1 min at UV365 nm, as well as aristolochic acid I and II (Rf 0.46 and Rf 0.54) [18].

#### 2.1.2. *Magnolia officinalis* Rehder & E.H. Wilson

Alkaloid and lignan detection was made using HPTLC-MS. As described by Estevez et al., mass spectrometry can indeed be used to characterize and identify components from toxins [27].

##### Alkaloids

Following several protocols, the most appropriate mobile phase was selected for the *M. officinalis* extracts.

Each plate was revealed using a Draggendorff reagent. We found that, following Japanese Pharmacopoeia XVI, the most suitable mobile phase composition was butanol/water/acetic acid (at a proportion of 4/2/1).

##### Lignans

According to European Pharmacopoeia 8.4, a methanol/ethyl acetate/toluene mobile phase (4/8/120, *v*/*v*/*v*) was used and observed using vanillin reagent.

Both lignans, magnolol and honokiol, and almost 15 different alkaloids were detected in methanolic and aqueous extracts (see Table 1).

### 2.2. Cytotoxicity of the Plant Extract Alone or in Combination

**Figure 1:**
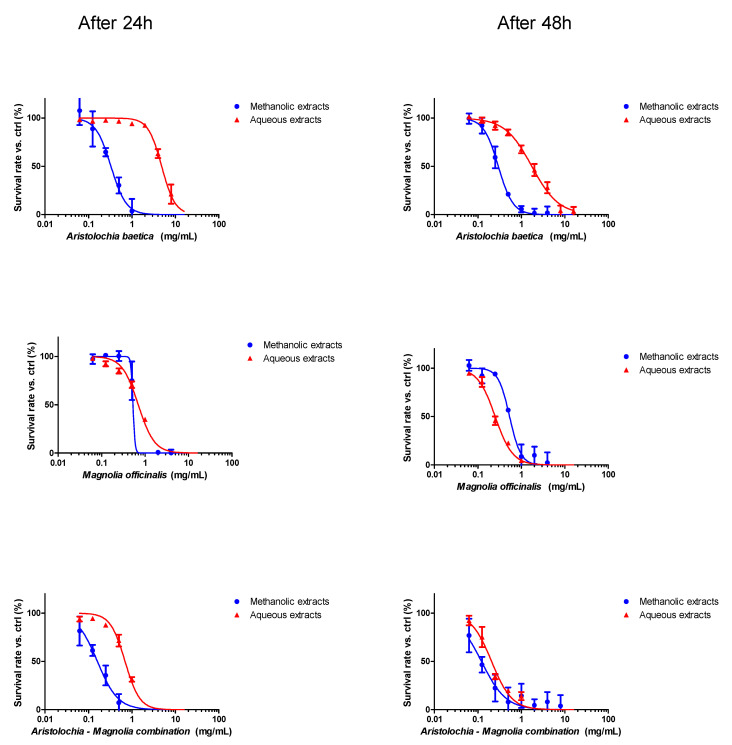
Resazurin assay for HK-2 cell survival evaluation after incubation for 24 h and 48 h with methanolic or aqueous extracts of *Aristolochia baetica* or *Magnolia officinalis*, respectively, alone or in combination (means +/− SD from three separate experiments in duplicate (n = 6)).

The cytotoxicity of *Aristolochia* and *Magnolia* aqueous and methanol extracts was investigated on HK-2 cells by following resazurin viability curves after 24 and 48 h of exposure to each material alone, or in combination. Results are presented in Figure 1 and Table 2.

One-way ANOVA (Dunnett’s multiple comparison test; *p* < 0.01; n = 6) confirmed that the viability more significantly decreased with the *Magnolia* than the *Aristolochia* extracts, after 24 and 48 h of exposure. The combination of both plant extracts resulted in a more significant deleterious effect on cell viability.

### 2.3. Genotoxicity throughout γ-H2AX Detection

**Figure 2:**
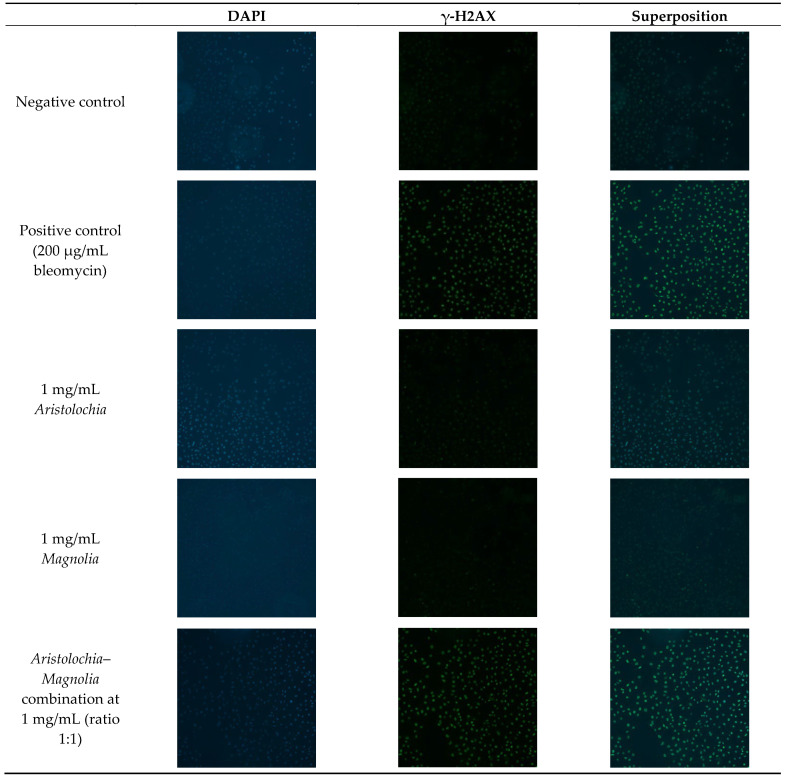
Representative immunofluorescence images obtained from control HK-2 cells and cells exposed for over 24 h to aqueous plant extracts alone or in combination, and to 200 µg/mL bleomycin (20x magnification, 1 cm = 500 µm, n = 4).

The measurement of the phosphorylated histone γ-H2AX is increasingly considered as an attractive biomarker for either DNA damage or DNA repair. H2AX is a eukaryotic histone protein that, depending on the organism and cell type, constitutes 2–25% of the mammalian histone H2A. Interestingly, this histone is phosphorylated on its 139th serine residue in the presence of DNA damage, mainly a double-strand break, to yield γ-H2AX. The latter form nuclear domains, named “DNA damage foci”, can be visualized cytologically using fluorescence microscopy [18,24,25]. γ-H2AX detection by immunofluorescence in HK-2 cells demonstrated that treating cells with bleomycin for 24 and 48 h induced an increase in γ-H2AX foci at all tested concentrations: 50, 100, and 200 µg/mL (data not shown). By comparison with these positive controls, cells treated with the aqueous extract at a 1 mg/mL concentration of a *Aristolochia*–*Magnolia* combination (1:1 ratio) demonstrate an increase in γ-H2AX foci, while the *Aristolochia* or the *Magnolia* aqueous extracts used alone did not induce any significant increase in γ-H2AX foci (Figure 2). Cells treated with a combination of *Aristolochia* and *Magnolia* aqueous extracts at 0.25 and 0.5 mg/mL did not significantly increase the number of γ-H2AX foci (data not shown).

**Figure 3:**
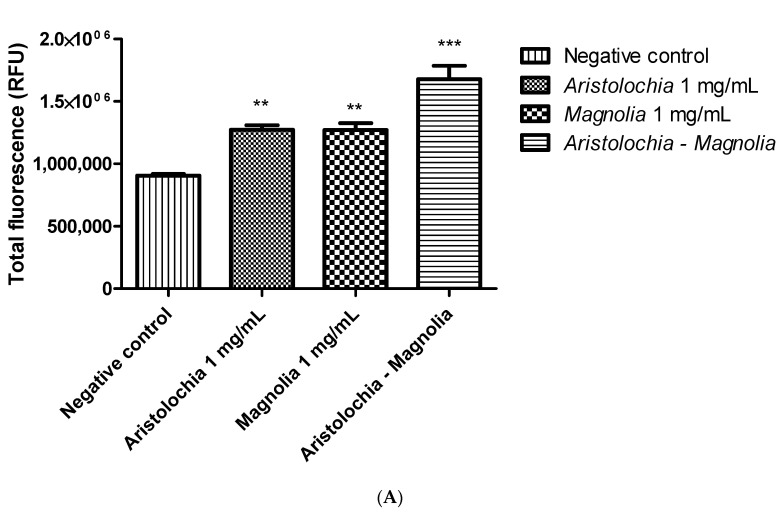

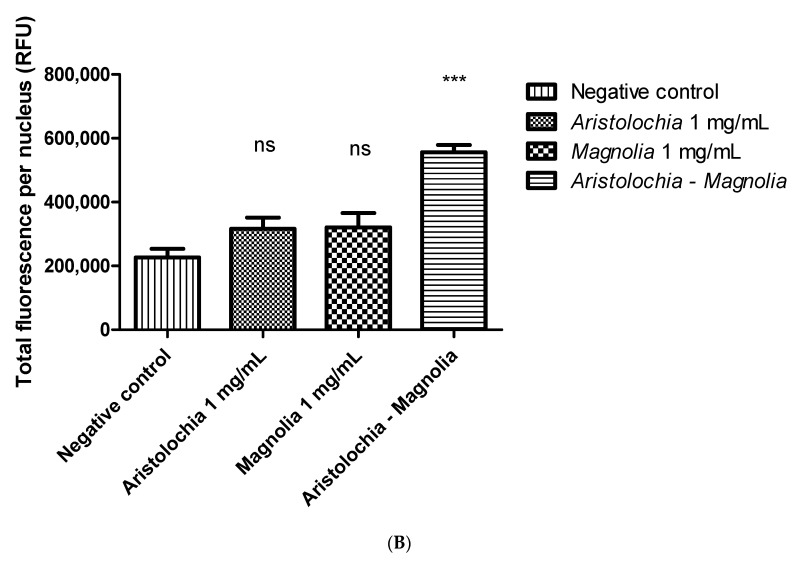
(**A**) Total fluorescence (RFU) and (**B**) total fluorescence per nucleus (RFU) after 24 h of HK-2 cells exposure (n = 4) to aqueous extracts of *Aristolochia* (1 mg/mL), *Magnolia* (1 mg/mL), and a *Aristolochia–Magnolia* combination at the same concentration (1:1 ratio), respectively. Each analysis corresponds to the 3000 microscope fields counted. A one-way ANOVA test was followed by Dunnett’s multiple comparison test with the control. ns: not significant vs. negative control. ** *p* < 0.01; *** *p* < 0.001.

γ-H2AX fluorescence detection has been quantified by measuring total fluorescence and total fluorescence relative to the number of nuclei. Statistical significance could not be demonstrated for extracts used alone. However, statistical significance could be highlighted by using a combination of the two plants (see Figure 3).

**Figure 4:**
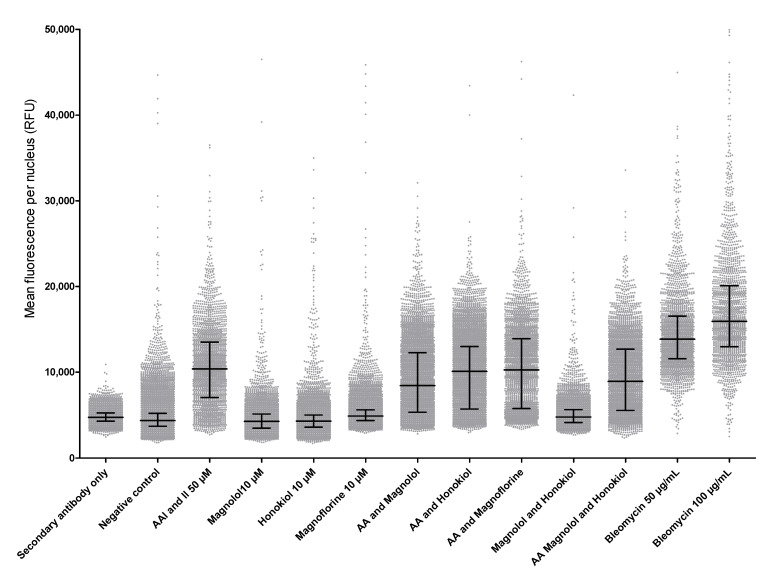
Mean fluorescence per nucleus of γH2AX after 24 h of HK-2 cell exposure (n = 3). The negative control represents untreated cells. Bleomycin is the positive control.

We further investigated which component could explain this increasing genotoxicity. We performed the same experiment with aristolochic acids (I and II), magnolol, honokiol, magnoflorin, and a combination of all of them over 24 h and 48 h exposure. Unfortunately, we did not observe any increase in the presence of γ-H2AX after these different experimental conditions (Figure 4—data not shown for 48 h).

### 2.4. Genotoxicity and Mutagenicity throughout the Ames Test

An Ames test was run using four strains of histidine-deficient *Salmonella typhimurium*: TA-100, TA-98, TA-1535, and TA-1537, with and without the S9 fraction. The number of revertants was recorded for each sample and compared to a positive control.

With the TA-100 strain, *A. baetica* seems to be mutagenic at every concentration from 0.25 mg/mL, without the S9 microsomal fraction. In the presence of the S9 fraction, only the concentration of 10 mg/mL was mutagenic. The TA-1535 strain, on its side, does not seem to be sensitive to *Aristolochia* or *Magnolia* extracts (data not shown). A statistically significant increase in the mutagenicity induced by *Aristolochia* extracts alone, as well as the *Aristolochia–Magnolia* combination, was found with the strain TA-98, but only with the S9 microsomial liver fraction. The TA-98 strains reveal frameshift mutations, meaning that the genotoxicity is based on a genetic mutation caused by the indels (insertions or deletions) of a number of nucleotides in a DNA sequence. Moreover, this mutagenic effect appears to be dose-dependent (Table 3).

## 3. Discussion

The bark of *M. officinalis* Rehder & Wilson, known under the pinyin name “Hou Po,” has been traditionally used in Chinese and Japanese medicines for the treatment of anxiety, asthma, depression, gastrointestinal disorders, and headaches. Many pharmacological activities, including antioxidant, anti-inflammatory, antibiotic, and antispasmodic effects [20], as well as its alleviating effect on depression in postnatal women [28], have been reported for this herb and its major compounds. Many of these activities have been attributed to the lignans magnolol and honokiol, two major constituents of the plant.

In the present in vitro study, we first demonstrated that the *M. officinalis* aqueous and methanolic extracts show a higher cytotoxicity after 24 and 48 h than the aqueous and methanolic extracts of *Aristolochia baetica*. Combining both plant extracts resulted in the most deleterious effects.

However, we were not able to identify which component association actually increased the generation of γH2AX foci. Therefore, it seems that lignans (i.e., Magnolol and Honokiol) and the tested alkaloid magnoflorin are not the agents responsible for the increased toxicity of *Aristolochia.* Considering the characterization of our *Magnolia officinalis* extracts, we can hypothesize that many other components could be candidates for these effects, mainly alkaloid compounds.

Concerning the mutagenic assessment of our extracts, we performed the Ames test. The Ames test is one of the most frequently applied tests in toxicology [29]. Following the OECD guidelines, almost all new pharmaceutical substances and chemicals used in the industry are tested by this assay [30]. The Ames test, or the so-called *Salmonella*/microsome test, is widely used in investigating the mutagenic effects of chemicals. Not only is it one of the most reliable short-term bacterial test systems, but it is also cheap and provides results rapidly [31,32]. Each herbal extract was tested on four different strains. TA100 and TA1535 were used to detect base substitution mutations, and TA98 and TA1537 were used to detect frameshift mutations. The liver S9 fraction is useful for imitating the mammalian metabolism that activates pro mutagens [26,31,32,33].

The *Salmonella* strain TA-98, in the presence of the S9 fraction, showed that the *Magnolia* and *Aristolochia* aqueous extract combination is more mutagenic than both aqueous extracts tested separately. This result suggests that the combination generates frameshift mutations and that this effect is dose-dependent. The need for the S9 fraction proves that the combination of plant extracts has to be metabolized by hepatic enzymes to be mutagenic. This pathway is related to the fact that AAN patients ingested root extracts as slimming pills. After oral intake, these pills, following the pharmacokinetic features, were metabolized by the liver before targeting the kidneys where they generated their toxicity.

Although AA might directly cause nephropathy, the enzymatic activation of AAI is required to exert its genotoxic effect. The reduction of the nitro group is now considered to be the major activation pathway of AA [16]. NAD(P)H:quinone oxidoreductase (NQO1) and microsomal enzyme NADPH:cytochrome P450 oxydoreductase are the main enzymes responsible for the metabolic activation of AA in aristolactams by reduction of the nitro group, and they may be involved in the bioactivation of AA, but its exact roles are still a matter of debate [16,34,35,36,37]. Interestingly, in 2013, Cui et al. showed in a mouse model that kidney NQO1 was significantly increased following treatment with an ethanolic extract of *Magnolia officinalis* bark [38].

It has been recently demonstrated, for the first time, that cell lines deficient in nucleotide excision repair (NER) machinery accumulated higher adduct levels, indicating that NER is the major mechanism responsible for the repair of these lesions [39].

Following our results, the high potentiation of AA genotoxicity by *Magnolia officinalis* could then be tentatively explained by an increased metabolic activation into aristolactams. More investigations are needed to determine which components could be responsible for the increase in DNA damage.

## 4. Conclusions

Plants are interesting from a health point of view but can be toxic. Moreover, these toxicities are not always direct but can occur months or years later. This further complicates the identification of the origin of these adverse reactions. This study demonstrated that *Magnolia officinalis*, often described as safe, seems to be relatively cytotoxic, mainly to kidney cells, but also seems to have antibacterial activity (significantly cytotoxic and mutagenic to *Salmonella typhimurium* TA-100 strains at 10 mg/mL). Moreover, the genotoxicity to the kidney of the association of *Aristolochia baetica* and *Magnolia officinalis* extracts is significantly higher compared to the individual plant extracts. This was also confirmed by an Ames test, an OECD-recommended mutagenicity assessment assay. The high potentiation of AA genotoxicity by *M. officinalis* could be an explanatory factor for Chinese herb nephropathy cases observed in Belgium in the 1990s. Furthermore, plants are, in most cases, used in combination rather than alone. This study proves that plant combinations can be more toxic under specific conditions.

## 5. Materials and Methods

### 5.1. Herbal Extraction

As previously performed by Nachtergael et al. [18], dried *Magnolia officinalis* Rehder & E. H. Wilson cortices were obtained from Phytax (Schlieren, Switzerland). A certificate of analysis was obtained from the company, indicating a sample free from aflatoxins (detection limit: 0.4 µg/kg) and heavy metals (detection limit: 10 µg/kg). Instead of the *Aristolochia fangchi* Y. C. Wu ex L. D. Chow & S. M. Hwang from the initial prescription, now prohibited in Belgium, *Aristolochia baetica* L. radix was used in the present study. It is deposited at the National Herbarium of Morocco (Scientific Institute of Rabat) under the voucher name RAB 78463. Further investigations proved that *A. baetica* L. also contains quite similar aristolochic acid composition [18]. Each herbal extract has been prepared using polar solvents (water and methanol) (Table 4).

For both aqueous and methanolic extracts, the maximal concentration tested was 1 mg/mL. Plant combinations were assessed in a 1:1 ratio. Indeed, the initial prescription followed the typical formula: 100—200 mg of *Stephania* (adulterated by the roots of an *Aristolochia* species, probably *A. fangchi*), 100–200 mg of *Magnolia* (bark of *Magnolia officinalis* Rehder & E. H. Wilson), 2 mg of *Belladona* dry extract, 45 mg of acetazolamide, 20 mg of fenfluramine, 20 mg of diethylpropion, and 50 mg of meprobamate. The two plants were present in slimming pills at the same concentration, and this is why a 1:1 mixture was selected for use.

The common dosages for *Magnolia officinalis* and *Aristolochia* species are 3–9 g and 4.5–9 g, respectively [40]. A study showed that, following a 240 mL dose of water, the intestinal liquid volume ranges from 67 ± 17 to 93 ± 24 mL, respectively, 2 and 45 min after ingestion [41]. The liquid volume is divided into water pockets, where 60% of the total volume is contained in a small number of large pockets (>20 mL), and 40% is contained in a large number of the smallest pockets (0.5–20 mL). Regarding the 100–200 mg of plants ingested in the Belgian cohort patients, the concentration reached in the small intestine can be coarsely estimated at around 100–200 mg/100 mL, or 1–2 mg/mL, for each plant. The concentration used in the present study is in line with the estimated concentration of plants in the small intestine after the ingestion of slimming pills.

#### 5.1.1. Aqueous Extraction

Each material was decocted in water, following the traditional instructions in the *Chinese Materia Medica* [40]. The decoction was filtered on cellulose 3 times, and the filtrate was lyophilized (Heto PowerDry LL1500, Thermo Fisher Scientific Inc., Waltham, MA, USA) and stored at −20 °C until further use. The extracts were dissolved and diluted with a complete culture medium to the required concentrations.

#### 5.1.2. Methanolic Extraction

Following the Japanese Pharmacopoeia, 13th Edition (1996), each plant was macerated in a known volume of methanol for 24 h in the dark. Plants were mixed with a Polymix PX-MFC 90 D mixer (1.0 mm diameter). The solvent from the resulting mixture was harvested and removed from the extract by the rotary evaporator (Rotavapor R-210, Vacuum pump V-700, Vacuum controller V-850, Heating Bath B-491).

### 5.2. Extract Characterizations

The raw and lyophilized herbal materials and the methanolic extracts were analyzed by HPTLC thin-layer chromatography according to the European Pharmacopoeia 8.4 monographs “*Magnolia officinalis* bark” and “Test for aristolochic acids in herbal drugs.” Comparable amounts of raw herbs and extracts (calculated from the lyophilization yield) were applied on HPTLC plates (HPTLC silica gel 60 F254 glass plates).

*Magnolia officinalis* was also analyzed for alkaloids according to the Japanese Pharmacopoeia, 16th Edition (1996), using HPTLC-MS (Camag TLC-MS interface). Standards of aristolochic acid mixtures of AAI and AAII (HPLC purity: 98.7%; Acros Organics) were purchased from Thermo Fisher Scientific (Geel, Belgium). Magnolol (HPLC purity: 100.0%) and honokiol (HPLC purity: 100.0%) were purchased from Extrasynthèse (Genay, France). Magnoflorine was purchased from Sigma-Aldrich (reference SMB00377).

### 5.3. Cell Culture of HK-2 Kidney Proximal Human Tubule Cells

HK-2 cells, originating from human RPTECs, were obtained from American Type Culture Collection (CRL-2190, ATCC, Manassas, VA, USA), and grown in low-glucose DMEM containing 10% fetal bovine serum (FBS PAA Clone, PAA laboratories, Pasching, Austria), 2 mM L-glutamine, and 1% penicillin-streptomycin. Cells were sub-cultured or harvested for experiments when reaching about 90% confluence. For experimental purposes, cells were used between Passage 6 and 25, harvested by trypsinization, and seeded on 12-well plates (1 × 10^5^ cells), 8-well chambered slides (Lab-Tek II, Nunc, Rochester, NY, USA) (2 × 10^4^ cells), or 96-well plates (1 × 10^4^ cells). Next, cells were incubated for 24 h in an FBS-containing medium, rinsed twice with DMEM, and treated with test substances at a concentration from 0.0625 to 16 mg/mL in an FBS-depleted medium.

Preliminary cytotoxicity assays were performed by Bunel et al. [12] to determine the working doses of each compound. Based on their recognized cytotoxicity in vivo, AA was used at IC25 (≈50 µM); maximum concentrations of magnolol and honokiol were the highest non-lethal doses (10 µM).

Aristolochic acids were a 50:50 mixture of AAI and AAII (HPLC purity: 98.7%; Acros Organics, Thermo Fisher Scientific, Geel, Belgium). Magnolol (HPLC purity: 100.0%) and honokiol (HPLC purity: 100.0%) were purchased from Extrasynthèse (Genay, France). Magnoflorine was purchased from Sigma-Aldrich (reference SMB00377).

### 5.4. Cell Viability Assay by a Resazurin Assay

As described by Bunel et al. [12], wells were treated with test compounds in 96-well plates, washed twice with PBS, and assessed for their viability (metabolic activity) by incubation with 0.44 mM resazurin solution (Sigma-Aldrich, St. Louis, MI, USA) for 1.5 h at 37 °C. Absorbances were measured at wavelengths 540 and 620 nm and percentages of reduced dye were calculated with the following formula:

$$\frac{\left(\varepsilon \mathrm{OX}\right)\lambda 2\cdot A\lambda 1-\left(\varepsilon \mathrm{OX}\right)\lambda 1\cdot A\lambda 2}{\left(\varepsilon \mathrm{RED}\right)\lambda 1\cdot A’\lambda 2-\left(\varepsilon \mathrm{RED}\right)\lambda 2\cdot A’\lambda 1}$$

where εOX is the molar extinction coefficient of resazurin (47.6 at 540 nm and 34.8 at 620 nm); εRED is the molar extinction coefficient of resorufin (104.4 at 540 nm and 5.5 at 620 nm); A is the absorbance of the test wells; A’ is the mean absorbance of the blank wells; λ1 = 540 nm; λ2 = 620 nm. Metabolic activity was normalized against the control condition.

Absorbances were measured with an iEMS Reader MF spectrophotometer (Thermo Labsystems, Breda, The Netherlands). The positive control (bleomycin, 0.8 to 200 µg/mL) or negative control (supplemented medium) was added.

### 5.5. IF 96-Well on the HK-2 Kidney Cell Line

Cells were seeded in 96-well plates (10,000 cells per well) and grown at 37 °C for 24 h before plant extracts, AA, lignans (both or separately), magnoflorine, or a positive (bleomycin, 200 µg/mL) or negative control (supplemented medium) were added.

After 24 h or 48 h, the cells were washed with PBS for 5 min, fixed with 100 µL/well of 4% PFA for 20 min, washed 4 times consecutively with tap water, distilled water, PBS with triton (0.01%), and PBS alone (100 µL/well) for 5 min. The cells were incubated overnight at 4 °C with 100 µL/well of a 1/1000 dilution of the anti-γ-H2AX primary antibody (goat anti-serum, 20%) for 1 h at room temperature. The cells were washed with 100 µL/well PBS twice. Cells were then incubated overnight on a mild rotative shaker at 4 °C with 30 µL/well of a 1/1000 dilution of the DyLight 488 conjugated secondary antibody (Mouse Phospho-Histone H2A.X pSer140 Antibody (3F2)-species reactivity: mouse, human, and bovine, Thermo Scientific^®®^ ref MA1-2022) in goat serum, washed with PBS twice for 5 min (100 µL/well).

Work henceforth progressed in the dark. Cells were mounted using a few microliters of ProLong mounting medium with DAPI. A coverslip was added (Cover Glasses, 6 mm diameter, thickness n.0 VWR-Cat. 631-0168), and plates were recovered by adhesive film for microplates (VWR-Ref. 391-1254). Plates were conserved in aluminum at 4 °C.

The fluorescence was quantified using a microscope Axio Observer Z.1 (Zeiss) and Image J.

### 5.6. Bacterial Reverse Mutation Test or Ames Test

An Ames test was run using five strains of histidine-deficient *Salmonella typhimurium*—TA-100, TA-98, TA-1535, and TA-1537—with and without the liver S9 fraction. This liver S9 fraction is used to imitate mammalian metabolism [26]. The Ames test is one of the most frequently applied tests in toxicology. Almost all new pharmaceutical substances and chemicals used in industry are tested by this assay. Each herbal extract was tested on four different strains. TA100 and TA1535 were used to detect base substitution mutations, and TA98 and TA1537 were used to detect frameshift mutations. This strain was purchased from Xenometrix (Anaria) (Ames MPF^TM^ 98/100/1535/1537 kit). The positive controls used were 2-nitrofluorene (2 µg/mL, Xenometrix^®^ (AA01-410)), *N*^4^-aminocytidine (100 µg/mL, Xenometrix^®^), 4-nitroquinoline-*N*-oxide (0.1 µg/mL, Xenometrix^®^), and 9-aminocridine (15 µg/mL, Xenometrix^®^) (Table 5).

Sample concentrations assayed were from 0.05 to 10 mg/mL. The mutagenic effect of different herbal extracts was assessed after 48 h of incubation with the 4 strains (TA98, TA100, TA1535, and TA1537) in triplicate, with and without the S9 fraction.

A t-test was performed (*p*-values, 1-sided, based on unpaired data). The level of significance was *p* ≤ 0.05.

## 6. Statistical Analysis

Statistical analysis was performed with GraphPad Prism 5 software (GraphPad Software, La Jolla, CA, USA). A probability level of *p* < 0.05 was considered significant.

## Figures and Tables

**Table 1:** Alkaloids (mainly benzylisoquinoleine alkaloids) and lignans detected in barkaqueous and methanolic extracts of *M. officinalis*, respectively, by the TLC-MS negative ESI mode for lignan detection and the positive ESI mode for alkaloid detection.

Molecules	MS Detection (*m*/*z*)	Aqueous Extract	Methanolic Extract
Alkaloids			
Anaxagoreine	284 (M + H) ^+^ 283 + D ^+^	yes	yes
Asimilobine	268 (M + H) ^+^	yes	no
(*S*)-4-Kétomagnoflorine	356 (M) ^+^	yes	yes
Corytuberine	328 (M) ^+^	yes	yes
1,9,10-Trihydroxy-2-méthoxy-6,6-diméthylaporphinium			
Liriodénine	276 (M + H) ^+^	yes	yes
Lotusine	314 (M) ^+^	yes	yes
(*R*)-Magnocurarine			
(*R*)-Oblongine			
(*S*)-Magnoflorine	342 (M) ^+^	yes	yes
*N*-Méthylcoclaurine	300 (M + H) ^+^	yes	yes
Remerine	315 (M + H) ^+^	yes	yes
Réticuline	330 (M + H) ^+^	yes	yes
(*S*)-Tembetarine	344 (M) ^+^	yes	yes
Lignans			
Magnolol	265 (M + H) ^−^	yes	yes
Honokiol	265 (M + H) ^−^	yes	yes

**Table 2:** Cell viability was determined by the resazurin assay on HK-2 cells. Curves have been fitted to the data from three separate experiments (n = 6) and have been fitted with nonlinear regression (log(inhibitor) vs. normalized response—variable slope).

	Resazurin Assay and IC50			
	Aqueous Extracts		Methanolic Extracts	
	After 24 h	After 48 h	After 24 h	After 48 h
*Aristolochia baetica*	4.8 mg/mL	1.7 mg/mL	0.3 mg/mL	0.3 mg/mL
*Magnolia officinalis*	0.7 mg/mL	0.2 mg/mL	0.5 mg/mL	0.5 mg/mL
*Aristolochia–Magnolia* (1:1)	0.7 mg/mL	0.2 mg/mL	0.2 mg/mL	0.1 mg/mL

**Table 3:** Results of the Ames tests performed on *Salmonella typhimurium* strains TA-98 and TA-100 with and without the S9 fraction, after exposure to aqueous extracts of *Aristolochia, Magnolia*, or a combination of both plants. Baseline = Mean ± 1 SD. A t-test was performed (*p*-values, 1-sided, based on unpaired data). Level of significance: *p* ≤ 0.05 (n = 3). Values ≥ 2.0 are considered to be positive (mutagenic).

Dose (mg/mL)	Baseline	Fold Increase over the Baseline	Significant Increase Compared to Concurrent Vehicle Control (*t*-test)
TA-98 strain			
*Aristolochia*—S9 activation			
0	1.24		
10		0.80	0.3217
5		3.48	0.0212
1		2.41	0.0124
0.5		1.61	0.0581
0.25		1.07	0.1151
0.05		0.27	0.2593
*Magnolia*—S9 activation			
0	5.55		
10		0.24	0.2383
5		0.66	0.3120
1		1.14	0.0745
0.5		1.08	0.1072
0.25		0.66	0.3036
0.05		0.36	0.3744
*Aristolochia–Magnolia* combination—S9 activation			
0	1.24		
10		4.29	0.0300
5		3.75	0.0163
1		5.09	0.0095
0.5		2.14	0.0066
0.25		0.54	0.5000
0.05		1.61	0.0581
TA-100 strain			
*Aristolochia*—S9 activation			
0	4.00		
10		2.17	0.0029
5		1.67	0.0127
1		1.33	0.0286
0.5		0.75	0.5000
0.25		0.58	0.3351
0.05		0.92	0.2459
*Aristolochia*—Without S9 activation			
0	1.91		
10		3.84	0.0016
5		3.66	0.0000
1		2.44	0.0121
0.5		2.27	0.0079
0.25		1.92	0.0176
0.05		0.52	0.3217

**Table 4:** Aqueous and methanolic extraction yields.

Plants	Pictures	Aqueous Decoction Time (min)	Lyophilization Yields	Methanolic Extraction Yields
*Magnolia officinalis* Rehder & E.H. Wilson Cortex	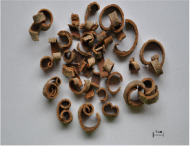	15	1.5%	12.1%
*Aristolochia baetica* L. Radix	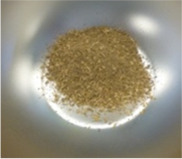	45	23.4%	23.5%

**Table 5:** *Salmonella thyphimurium* strains and parameters detected by the Ames test.

Strain	Mutation	Mutation Detected	Target	Positive Control
TA98	*his*D3052	Frameshifts	GCGCGCGC	2-nitrofluorene (2-NF)
TA100	*his*G46	Base–pair substitution	GGG	4-nitroquinoline *N*-oxide (4-NQO)
TA1535	*his*G46	Base–pair substitution	GGG	*N*^4^-aminocytidine (N^4^-ACT)
TA1537	*his*C3076	Frameshifts	+1 frameshift (near C-C-C run)	9-aminocridine (9AA)

## Data Availability

Not applicable.
